# Effects of Pre-Tensile Deformation on the Fatigue Fracture Behavior of Annealed 7005 Aluminum Alloy Plate

**DOI:** 10.3390/ma15020623

**Published:** 2022-01-14

**Authors:** Ni Tian, Zhen Feng, Xu Shi, Wenze Wang, Kun Liu, Gang Zhao, Gaowu Qin

**Affiliations:** 1 School of Materials Science & Engineering, Northeastern University, No. 3-11, Wenhua Road, Heping District, Shenyang 110819, China; 2070348@stu.neu.edu.cn (Z.F.); 1900590@stu.neu.edu.cn (X.S.); 2070509@stu.neu.edu.cn (W.W.); zhaog@mail.neu.edu.cn (G.Z.); qingw@smm.neu.edu.cn (G.Q.); 2 Key Laboratory for Anisotropy and Texture of Materials (Ministry of Education), Northeastern University, No. 3-11, Wenhua Road, Heping District, Shenyang 110819, China; 3 Department of Applied Science, University of Quebec at Chicoutimi, 555, Boul. de L’university, Chicoutimi, QC G7H2B1, Canada; kun.liu@uqac.ca

**Keywords:** annealed 7005 aluminum plate alloy, pre-tensile deformation, dislocation, fatigue

## Abstract

In the present study, the fatigue life and fatigue fracture characteristics of annealed 7005 aluminum alloy plates subjected to different pre-tensile deformations were investigated. The results obtained upon increasing the pre-tensile deformation of the alloy plate to 20% revealed that the second-phase particles did not show any obvious changes, and that the thickness of the thin strip grain slightly decreased. The dislocation distribution in the alloy matrix varied significantly among the grains or within each grain as the dislocation density gradually increased with increasing pre-tensile deformation. Moreover, the fatigue performance of the annealed 7005 aluminum alloy plate was significantly improved by the pre-tensile deformation, and the alloy plate subjected to 20% pre-tensile deformation exhibited an optimal fatigue life of ~1.06 × 10^6^ cycles, which was 5.7 times and 5.3 times that of the undeformed and 3% pre-stretched alloy plates, respectively. Two fatigue life plateaus were observed in the pre-tensile deformation ranges of 3–5% and 8–12%, which corresponded to heterogeneous dislocation distribution among various grains and within each grain, respectively. Moreover, two large leaps in the plot of the fatigue-life–pre-tensile-deformation curve were observed, corresponding to the pre-tensile deformation ranges of 5–8% and 16–20%, respectively.

## 1. Introduction

The 7005 aluminum alloy, which has high specific strength, decent weldability, and hot working performance, is mostly used for structural profiles such as containers, storage tanks, bullet train and bicycle frames [1,2,3]. These structural materials are occasionally subjected to pre-deformation, which causes microstructural changes, during production, welding, and assemblage. Furthermore, pre-deformation considerably influences the micro-plasticity of materials under cyclic loading conditions, which can directly determine the fatigue life of materials [4,5,6]. However, different perspectives regarding the influence of pre-deformation on the fatigue properties of metallic materials have been presented.

A few reports have suggested that pre-deformation or pre-straining is detrimental to the fatigue performance and fatigue life of metallic materials. Chiou et al. [7] found that the cyclic softening effect during the fatigue loading process of SUS 430 stainless steel increased with an increase in the pre-tensile deformation, which significantly shortened its fatigue life under symmetric tension and compression fatigue loads as the pre-tensile deformation increased from 5% to 12%. Sun [8] revealed that pre-straining negatively affected the fatigue life of CP800 steel. Brancoa et al. [4] reported that the heterogeneous distribution of dislocations, such as dislocation stacking at slip bands and at grain boundaries, due to the pre-tensile deformation of the 7050-T6 aluminum alloy accelerating the damage accumulation at the grain boundaries, and reduced its fatigue life after pre-tensile deformations of <8%. Froustey et al. [9] found that the residual fatigue life of the 2017A-T3 aluminum alloy was significantly shortened with an increase in the pre-tensile deformation. White et al. [10] reported that a pre-tensile deformation of 1% was detrimental to the fatigue life of a friction-stir-welded joint of the AA7050 alloy. Al-Rubaie et al. [11] found that the sliding grains and persistent slip bands (PSBs) induced by pre-straining deformations of 1–7% were detrimental to the fatigue life of a 7050-T7451 aluminum alloy plate, and the reduction in fatigue life increased with the pre-straining level.

On the other hand, certain other reports have indicated that pre-deformation exhibits different effects on the high cycle fatigue (HCF) and low cycle fatigue (LCF) properties of metallic materials. Uemura [12] found that pre-deformation reduced the LCF life of mild steel but negligibly influenced its medium-to-high cycle fatigue. Kim et al. [13] reported that the yield strength and HCF performance of Fe-18Mn TWIP steel were significantly improved by pre-straining. However, the elongation and LCF strength of steel were found to decrease with pre-straining. Moreover, the LCF and HCF resistances decreased and increased, respectively, with an increase in the pre-strain deformation. Ghorbanpour et al. [14] found that pre-straining extended the HCF life of the WE43-T5 magnesium alloy at low stress amplitude but was detrimental to the LCF life at high stress amplitude. Park et al. [15] found that the dislocation strengthening caused by a pre-tensile deformation of less than 10% improved the yield strength of an AZ31 alloy plate but did not reduce its LCF strength. Arora and Raghavan [16] studied the influence of pre-tensile deformation on the rotational bending fatigue strength of the Al-0.93Si-0.60 Mg aluminum alloy. They found that the fatigue strength decreased by 7% when the aluminum alloy was pre-stretched by 1.5%. Moreover, the fatigue strength was close to that of the virgin material at higher pre-tensile deformations of 3–4%. In summary, the influence of pre-tensile deformation on the fatigue properties or fatigue life of metallic materials remains unclear. Additionally, the influence of pre-tensile deformation on the fatigue property or fatigue life of deformed 7005 aluminum alloy has not been extensively reported.

In the present study, a certain number and configuration of dislocations were generated in an annealed 7005 aluminum plate by uniaxial tensile deformation at room temperature. The influence of pre-tensile deformation on the static and dynamic mechanical properties of the alloy plate was investigated to reveal the strengthening effect of dislocation density, its distribution in the aluminum alloy matrix, and the synergistic action of the dislocation configuration and grain boundaries on the initiation and propagation of fatigue cracks in the 7005 aluminum plate. This would provide experimental data and a way to improve the fatigue performance of 7005 aluminum alloy plate.

## 2. Materials and Methods

**Figure 1:**
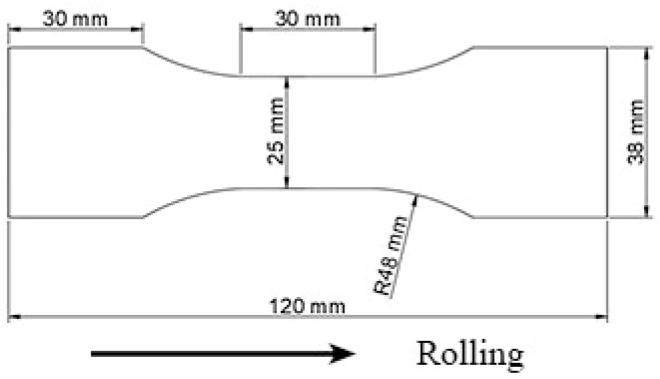
Dimensions of the 12.5 mm-thick tensile and fatigue specimens.

**Figure 2:**
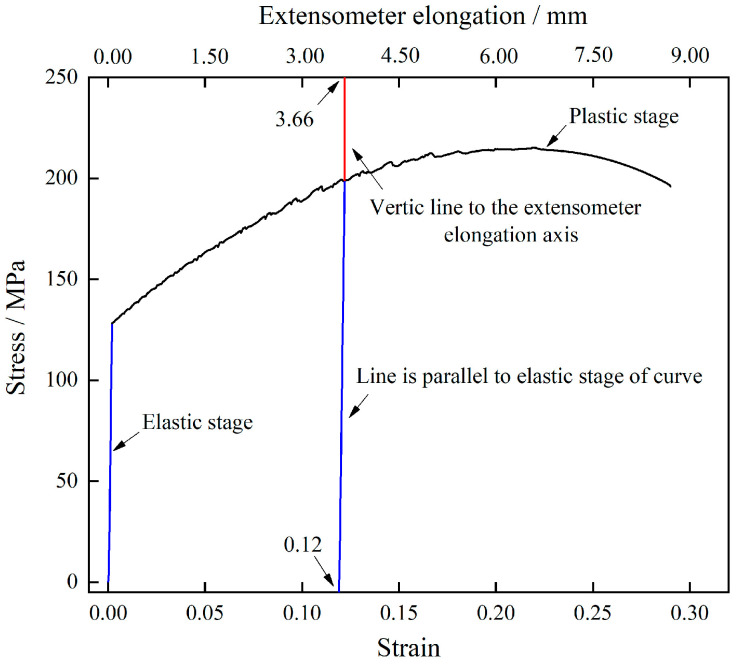
Tensile stress–strain curve of annealed alloy plate and schematic of pre-tensile deformation controlled by a large range extensometer.

Industrially produced 12.5 mm-thick 7005 aluminum alloy plates were used in this study, whose composition was as follows (wt.%): 4.67 Zn, 1.04 Mg, 0.13 Cu, 0.28 Mn, 0.17 Zr, 0.17 Cr, 0.132 Fe, 0.029 Si, <0.1 others, and balance Al. The 7005 aluminum alloy used in the present study was a commercially finished hot-rolled plate. They were DC casted and homogenized at 470 °C × 24 h followed by a double-roll hot rolling at 420 °C and a final rolling at 300 °C. The tensile and fatigue specimens were cut from the alloy plate along the rolling direction, and the loading direction of tensile and fatigue tests is consistent with the rolling direction, their specific dimensions are shown in Figure 1. All the specimens were annealed at 470 °C for 90 min in a resistance furnace and subsequently allowed to cool in the furnace for 24 h in order to have a similar initial state before pre-tensile deformation, such as grain structures, size and distribution of secondary particles. The tensile experiments were conducted using a Shimadzu AG-X 250 KN electronic universal testing machine (Shimadzu Corporation, Kyoto, Japan) at room temperature (25 °C), a strain rate of 1 × 10^−3^ s^−1^, and a data acquisition frequency of 100 Hz. The load accuracy was 100 N, and the stress and strain were measured using a force transducer and extensometer, respectively. Figure 2 shows the tensile stress–strain curve of annealed 7005 alloy plate and schematic of pre-tensile deformation controlling by a large range extensometer. The elongation of alloy plate correspond to different deformations can be measured by the extensometer elongation. For example, the 12% pre-stretched deformation can be controlled by the following steps: first, from the complete stress–strain curve, draw a parallel line from 0.12 point at the strain axis to the elastic stage of the stress–strain curve. Second, from the point of intersection of parallel line and stress-strain curve, draw a vertical line to the extensometer elongation axis. Third, the point of intersection of the vertical line and extensometer elongation axis is the control elongation for 12% pre-stretched deformation. The fatigue experiments were conducted using a QBG-100 high-frequency fatigue testing machine (Sinotest Equipment Co. Ltd., Changchun, China) at room temperature. A fatigue loading stress of 155 MPa, stress ratio (R) of 0, and a sine wave as the loading wave were used. Three repeat specimens and five repeat specimens were used for the tensile experiments and fatigue experiments, respectively.

Microhardness tests were conducted using a Wilson MH-500 digital display Vickers hardness testing machine (Buehler, IL, USA). An experimental load of 25 g and loading time of 10 s were used. Ten points on the rolling plane of alloy plate surface were measured in each section during the microhardness test to obtain an average value. The grain structure observation samples were anodic coated with film at a voltage of 25 V, and the composition of the coating solution was 1% HF + 1% HBF_4_ + 24% C_2_H_5_OH + 74% H_2_O. The microstructures of the central layer of the longitudinal section of the alloy plates were determined by optical microscopy (OM; OLYMPUS GX71, Olympus Corporation, Tokyo, Japan) and transmission electron microscopy (TEM; JEM-2100F, JEOL Ltd., Tokyo, Japan) at an accelerating voltage of 200 kV. Specimens for the TEM analysis were cut from the tensile specimen before and after different tensile deformations, thinned to approximately 90 µm, and subsequently electropolished in a twin-jet polishing unit (MTP-1A Magnetic Twin-jet Electropolisher, Shanghai Jiaoda Electromechanical Technology Development Co. Ltd., Shanghai, China). This unit was operated at 15 V and −25 °C using a 25% nitric acid and 75% methanol solution until perforation occurred. The fatigue fracture surface morphology was investigated by scanning electron microscopy (SEM; JSM-6510A, JEOL Ltd., Tokyo, Japan).

## 3. Results

### 3.1. Microstructure of Annealed 7005 Alloy Plate after Different Pre-Tensile Deformations

**Figure 3:**
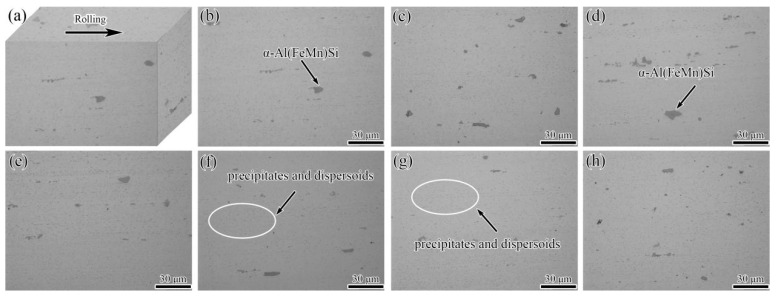
Optical microscopy (OM) images of the annealed 7005 aluminum alloy plate (**b**) before and (**c**–**h**) after different pre-tensile deformations (3%, 5%, 8%, 12%, 16%, and 20%, respectively, along the longitudinal section; non-etched), (**a**) shows a 3D diagram of the undeformed sample.

**Figure 4:**
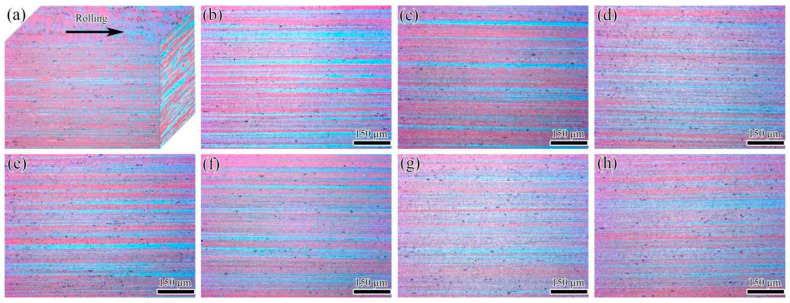
OM images of grains of the annealed 7005 aluminum alloy plate (**a**) before and (**b**–**h**) after different pre-tensile deformations (3%, 5%, 8%, 12%, 16%, and 20%, respectively, along the longitudinal section) acquired using a polarized light microscope, (**a**) shows a 3D diagram of the undeformed sample.

Figure 3 shows a three-dimensional (3D) stereogram-based metallographic microstructure and OM images of the longitudinal section of the alloy plate before and after pre-tensile deformations of 3–20%. Several micron-sized dark gray strips of second-phase particles were visible with fragmented chain distributions along the rolling direction, along with various second-phase particles that were fine, dispersed, light gray, and dotted in all of the alloy plates. Based on the results obtained by Hodgson [17] and Adrien [18], the regular or granular micron-sized dark gray strips of second-phase particles were presumed to be insoluble α-Al(FeMn)Si (as indicated by the arrow in Figure 3b), and the fine and dispersed light-gray dotted second-phase particles were primarily MgZn_2_ particles that were precipitated from α-Al during the annealing treatment of the alloy plate (as indicated by the circle in Figure 3f,g). No obvious differences were found in the shape, size, distribution, and quantity of the alloy phase particles between the aluminum plates subjected to the different pre-tensile deformations. Figure 4 shows a 3D diagram of the grains and OM images of the grains in the longitudinal section of the undeformed and pre-stretched (3–20%) 7005 aluminum plates. The grains of all the alloy plates were clearly observed to retain fibrous characteristics. The grains of the undeformed annealed alloy plate showed a thin strip shape (Figure 4a), indicating non-recrystallization in the alloy plate during the annealing treatment at 470 °C. The reason for non-recrystallization is mainly the pinning effect of the sub-micron dispersoid particles containing Mn, Cr and Zr on the migration of grain boundaries, which can raise the recrystallization temperature even retard the recrystallization. This is in accordance with the previously obtained results by Kazakova [19]. The thickness of the thin strip grains of the alloy plate decreased from 7.70 μm (undeformed specimen) to 5.02 μm (20%-pre-stretched specimen). In conclusion, a pre-tensile deformation of less than 20% did not have a significant effect on the shape, size, quantity, and distribution of alloy-phase particles in the annealed 7005 aluminum alloy plate. However, the thickness of the thin strip grains in the alloy plate slightly decreased with an increase in the pre-tensile deformation.

**Figure 5:**
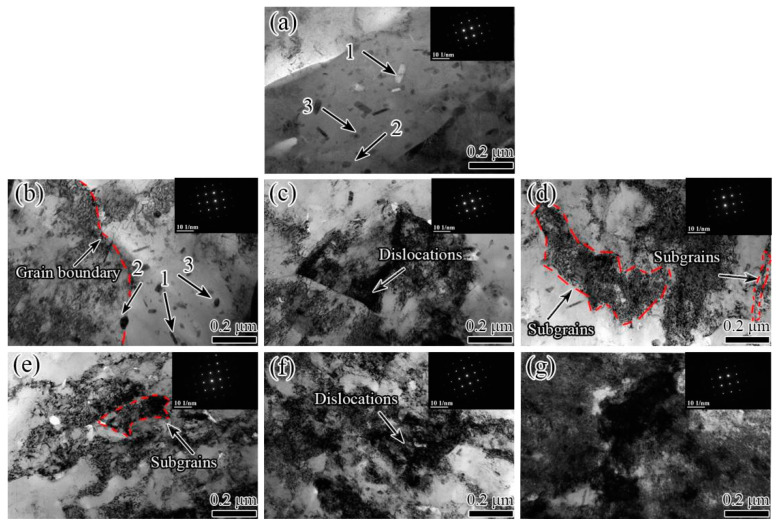
Bright-field transmission electron microscopy (BF-TEM) images of (**a**) undeformed and (**b**–**g**) pre-stretched annealed 7005 aluminum alloy plates (3%, 5%, 8%, 12%, 16%, and 20%, respectively).

**Figure 6:**
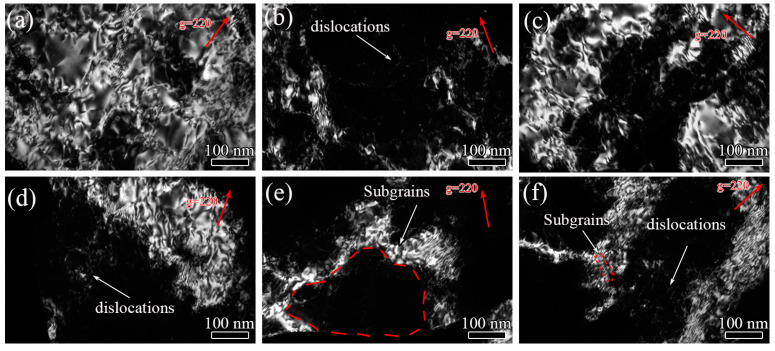
Dark-field transmission electron microscopy (DF-TEM) images of (**a**–**f**) pre-stretched annealed 7005 aluminum alloy plates (3%, 5%, 8%, 12%, 16%, and 20%, respectively).

Figure 5 shows BF-TEM images with the [001] zone axis indicated by the inserted diffraction pattern while Figure 6 displays DF-TEM images under diffraction vectors g = [220] of the 7005 aluminum alloy plates subjected to different pre-tensile deformations, respectively. Several sub-micron-sized second-phase particles were observed in the matrix of the alloy plates. Some of these second-phase particles were Mn-containing and Cr-containing dispersoids, whereas others were in the form of MgZn_2_ that was produced via precipitation from the matrix during the slow cooling process of annealing (Figure 5a, point 1, 2 and 3). No obvious differences were found in the shape, size, distribution, and quantity of the dispersoids and precipitates between the various pre-stretched aluminum plates. Only a few dislocations were observed in the undeformed annealed alloy plate matrix (Figure 5a). However, numerous dislocations were found in the different pre-stretched alloy plate specimens. Moreover, the dislocation density in the alloy matrix gradually increased and the dislocation distribution in the matrix showed significant differences between the various pre-stretched alloy plates with increasing pre-tensile deformation. At a pre-tensile deformation of <3%, the dislocation distribution in the alloy matrix was heterogeneous, and most of the dislocations were blocked at the grain boundary front (Figure 5b and Figure 6a). The dislocation density continued to increase with an increase in the pre-tensile deformation to 5%, and differences in the dislocation density in the grains were visible. This indicates that the dislocation distributions were heterogeneous in the different grains, and the dislocations accumulated in the vicinity of the grain boundaries in the yielding grains and formed a dislocation pile-up group plane, as shown in Figure 5c and Figure 6b. The phenomenon of inhomogeneous deformation and heterogeneous dislocation distribution in the pre-stretched alloy plates at a small deformation is consistent with the results obtained by Brancoa [4] for the 7075-T6 aluminum alloy matrix pre-stretched to less than 8%. The dislocation density in the alloy matrix further increased with an increase in the pre-tensile deformation to 8%. It is worth noting that the dislocation densities of the different oriented grains were nearly identical, and the dislocation distribution within each grain was homogeneous (Figure 5d and Figure 6c). The dislocation density continued to increase with increasing pre-tensile deformation to 12% and 16%, and the dislocation distribution within each grain became heterogeneous (Figure 5e,f and Figure 6d,e). The dislocation density in the grains and within each grain reached maximum values at a pre-tensile deformation of 20%. Moreover, the corresponding dislocation distribution was found to be almost homogeneous (Figure 5g and Figure 6f).

### 3.2. Mechanical Properties of Annealed 7005 Alloy Plates Subjected to Different Pre-Tensile Deformations

**Figure 7:**
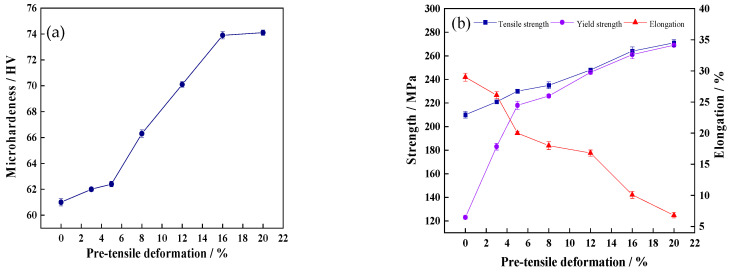
Mechanical properties of annealed 7005 aluminum alloy plates subjected to different pre-tensile deformations: (**a**) micro-hardness, (**b**) yield strength, tensile strength, and elongation.

The mechanical performance index curves of the annealed 7005 aluminum alloy plates subjected to different pre-tensile deformations are shown in Figure 7a,b. The microhardness, yield strength, and tensile strength of the alloy plate monotonically increased and the elongations gradually decreased with increasing pre-tensile deformation. The microhardness, yield strength, tensile strength, and elongation of the undeformed annealed alloy plate were 61.0 HV, 123 MPa, 210 MPa and 29%, respectively. The microhardness of the aluminum plates slowly increased up to a pre-tensile deformation of <5% and then linearly increased from 62.4 HV to 73.9 HV with increasing pre-tensile deformation from 5% to 16%. The 20%-pre-stretched alloy plate exhibited the maximum microhardness of 74.1 HV, which was 13.1 HV greater than that of the undeformed specimen. Furthermore, the yield strength of the alloy plates rapidly increased from 123 to 218 MPa at a pre-tensile deformation of <5%. Both the yield strength and tensile strength of the alloy plate linearly increased with a continuous increase in the pre-tensile deformation. The maximum yield strength and tensile strength of the 20% pre-stretched alloy plates were 269 MPa and 271 MPa, respectively, and the ratio of the yield strength to the tensile strength was greater than 0.99. These values were 146 MPa and 61 MPa higher than those of the undeformed annealed aluminum plate. This indicates that the annealed 7005 aluminum alloy plate exhibited a significant strain-hardening effect during tensile deformation at room temperature. Moreover, the influence of dislocation strengthening on the yield strength of the annealed 7005 aluminum alloy plate was significant, whereas that on the tensile strength of the alloy plate was relatively small.

### 3.3. Fatigue Life of Annealed 7005 Alloy Plates Subjected to Different Pre-Tensile Deformations

**Figure 8:**
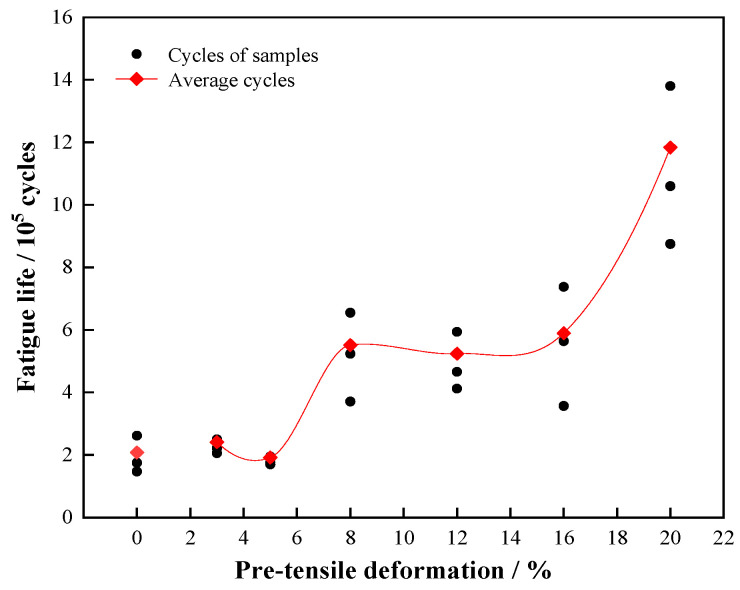
Fatigue life of annealed 7005 aluminum alloy plates subjected to different pre-tensile deformations (stress level, 155 MPa; stress ratio (R) = 0).

Figure 8 shows the fatigue life data of the different pre-stretched annealed 7005 aluminum alloy plates, a fatigue stress level of 155 MPa and an R value of 0 were used. It is worth noting that the yield strength of the undeformed annealed alloy plate was 123 MPa, and those of the pre-stretched alloy plates were all greater than 180 MPa; furthermore, the fatigue loading level of 155 MPa was higher than 123 MPa but less than 180 MPa. Therefore, the fatigue deformation behavior of the undeformed alloy plate was different from that of the pre-stretched aluminum alloy plates. The fatigue life of the annealed 7005 aluminum alloy plate was 1.86 × 10^5^ cycles, which was extended with increasing pre-tensile deformation. Two fatigue life plateaus were observed corresponding to the pre-tensile deformation ranges of 3–5% and 8–12%, respectively. In other words, the fatigue life of the alloy plates did not show obvious changes in the 3–5% (2.03 × 10^5^ cycles) or 8–16% (5.3 × 10^5^ cycles) pre-tensile deformation range. Additionally, two large leaps were observed in the fatigue-life–pre-tensile-deformation curve, corresponding to the pre-tensile deformation regions of 5–8% and 16–20%, respectively. Essentially, the fatigue life of the alloy plates was considerably extended in the aforementioned pre-tensile deformation regions. The 20% pre-stretched alloy plate had the longest fatigue life of ~1.06 × 10^6^ cycles, which was 5.7 times and 5.3 times those of the undeformed and 3%-deformed alloy plates, respectively. In conclusion, the pre-tensile deformation enabled a significant improvement in the fatigue properties of the annealed 7005 aluminum alloy plates.

### 3.4. Fatigue Fracture Morphologies of Annealed 7005 Alloy Plates Subjected to Different Pre-Tensile Deformations

**Figure 9:**
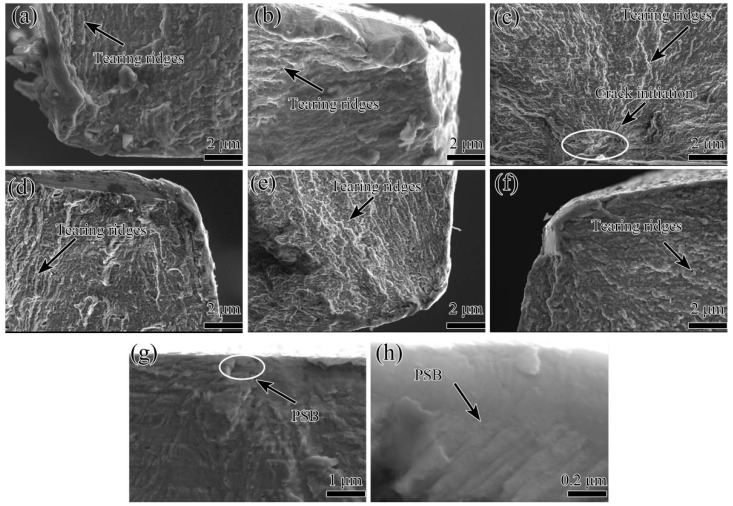
Scanning electron microscopy (SEM) images of fatigue fracture initiation in annealed 7005 aluminum alloy plates subjected to different pre-tensile deformations: (**a**) 3%, (**b**) 5%, (**c**) 8%, (**d**) 12%, (**e**) 16%, and (**f**) 20%, (**g**) PSB in 20% pre-stretched alloy plate, (**h**) local enlarged image of the circle zone in (**g**).

**Figure 10:**
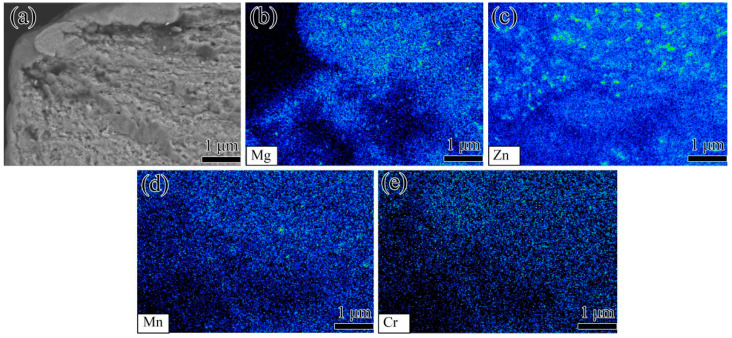
WDS images of fatigue fracture initiation in annealed 7005 aluminum alloy plate subjected to 3% pre-tensile deformation: (**a**) fatigue fracture initiation, (**b**) Mg element, (**c**) Zn element, (**d**) Mn element, (**e**) Cr element.

Figure 9 shows the morphologies of fatigue fracture initiation in the pre-stretched annealed 7005 aluminum alloy plates at a fatigue stress level of 155 MPa and at R = 0. The fatigue cracks in all the pre-stretched alloy plates were found to be initiated in the PSB at the surfaces or near the surfaces of the specimens, which is in accordance with the results previously obtained by Luo [20]. The fatigue cracks propagated radially from the fatigue crack initiation site deep into the interior of the alloy plates. The fatigue crack initiation sites of all the pre-stretched alloy plates were almost identical, implying that the increase in pre-tensile deformation did not significantly affect the fatigue crack initiation sites in the alloy plates. In addition, several rugged crystallographic planes and tearing edges were visible on the fatigue fracture crack initiation surfaces of the pre-stretched alloy plates, indicating changes in both the magnitude and orientation of the fatigue cracks during their propagation. The characteristics of the fatigue fracture morphology in the fatigue crack initiation region of the 3% and 5% pre-stretched alloy plates were almost identical. Moreover, the fracture surface significantly fluctuated, and the cleavage plane area was large (Figure 9a,b). The fatigue-cracked surface in the fatigue crack initiation region was smooth when the pre-tensile deformation of the alloy plates increased to 8%. Moreover, a small fracture surface fluctuation and a small cleavage plane area were observed. In other words, the spacing between adjacent tearing edges decreased (Figure 9c). However, the fluctuation of the fracture surface and the cleavage plane area in the fatigue crack initiation region increased slightly as the pre-tensile deformation increased to 12% and 16% (Figure 9d,e). In the 20% pre-stretched alloy plate, a rough fracture surface in the fatigue crack initiation region containing numerous fine and undulating cleavage planes was visible (Figure 9f), and PSB was observed near the surface of the specimen (Figure 9g,h). Figure 10 shows the BSE image and WDS images in the fatigue crack initiation region of the 3% pre-stretched alloy plates. There were many Mn-containing, Cr-containing, Zr-containing dispersoids and MgZn_2_ particles with homogeneous distribution in the fatigue crack, and no significant particle-induced fatigue cracks were observed in the fatigue crack initiation region. Therefore, it can be deduced that the fatigue crack initiations in all of the pre-stretched alloy plates were induced by PSBs on the specimen surface, not by the second-phase particles, by comprehensively analyzed Figure 9 and Figure 10.

**Figure 11:**
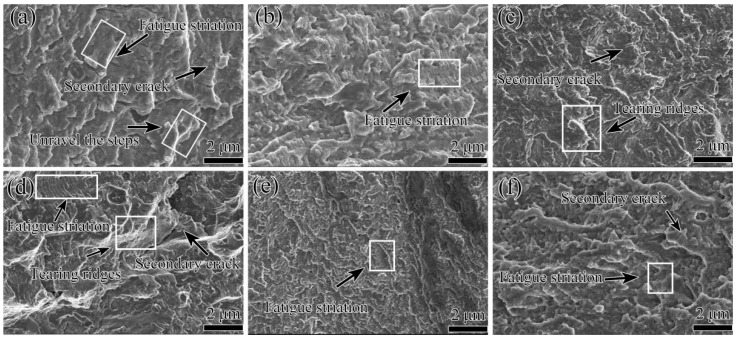
SEM images of fatigue fracture crack propagation in annealed 7005 aluminum alloy plates subjected to different pre-tensile deformations: (**a**) 3%, (**b**) 5%, (**c**) 8%, (**d**) 12%, (**e**) 16%, and (**f**) 20%.

Figure 11 shows the fatigue fracture crack propagation surfaces of the different pre-stretched annealed 7005 aluminum alloy plates; a fatigue stress level of 155 MPa and an R value of 0 were used. These results are very similar to those on the fatigue fracture crack initiation surfaces, as shown in Figure 9. Several rugged crystallographic planes and tearing edges were visible on the fatigue fracture crack propagation surfaces of the pre-stretched alloy plates, indicating that the direction of the fatigue cracks in the different pre-stretched alloy plates was deflected to different degrees during crack propagation. Other than the morphological characteristics of the fatigue fracture initiation surfaces of the alloy plates, typical fatigue striations, fatigue steps, and secondary cracks were observed on the fatigue fracture crack propagation surfaces that were distant from the fracture crack initiation sites. As the fatigue cracks continued to spread from the surfaces into the interior of the alloy plates, the stress concentration at the crack front and the fatigue crack growth rate both increased with increasing crack length. Furthermore, typical fatigue striations corresponding to a longer fatigue crack propagation length in a fatigue cycle were clearly observed.

However, differences in the surface morphological characteristics of the fatigue fracture propagation were clearly observed in the different pre-stretched alloy plates. Large wave-like crystallographic planes were observed in the fracture crack propagation zone of the 3% pre-stretched alloy plate, and significant surface fluctuation was found with large fatigue steps and a fatigue striation space of ~0.98 μm (Figure 11a). This suggests that the fatigue crack propagation direction in the 3% pre-stretched alloy plate was significantly deflected. However, the deflection frequency was low, indicating the deflection of fatigue fracture propagation between grains. The propagation direction of the fatigue cracks was presumed to primarily involve deflection between the grains. In the 5% pre-stretched alloy plate, although the surface fluctuation was considerable, the fatigue steps were larger and the area of the wave-like crystallographic planes was reduced. Moreover, the fatigue striation space diminished to 0.84 μm (Figure 11b). The increase in the deflection degree of the fatigue crack propagation and the strain strengthening of the matrix resulted in a decrease in the fatigue striation space in the 5% pre-stretched alloy plate. The fatigue fracture surface became smoother, and the surface fluctuation and the fatigue steps were smaller. However, the wave-like crystallographic planes did not show any significant changes, and the fatigue striation space further decreased to 0.78 μm. Moreover, certain small secondary cracks appeared in the fracture crack propagation zone when the pre-tensile deformation of the alloy plate was increased to 8% (Figure 11c). This indicates that the deflection degree of the fatigue crack propagation direction in the 8% pre-stretched alloy plate significantly decreased. However, the deflection frequency increased. Several small fatigue crack deflections within each grain were presumably generated during the crack propagation in the 8% pre-stretched alloy plate. At a higher pre-tensile deformation of the alloy plate of 12%, the surface fluctuation, fatigue steps, and area of the wave-like crystallographic planes, increased slightly. However, the fatigue striation space reduced to 0.64 μm (Figure 11d). In the alloy plate pre-stretched to 16%, the fatigue fracture surface became considerably smoother. Furthermore, the surface fluctuation and the fatigue steps were extremely small, the wave-like crystallographic planes were clearly diminished, and the fatigue striation space was further reduced to 0.52 μm (Figure 11e). This indicates that the deflection degree of the fatigue crack propagation direction significantly decreased, the number of deflections sharply increased, and the length of the fatigue crack expansion in one fatigue cycle was considerably reduced for the 16% pre-stretched alloy plate. A further increase in the pre-tensile deformation to 20% resulted in an extremely rough fatigue-fractured surface and a considerably fine fatigue striation space. Moreover, several fine and fluctuating spray-like crystallographic planes were visible on the fatigue crack propagation surface (Figure 11f). This is consistent with the previously observed rough morphology of the fatigue crack propagation surface in 7085, 7175, and 7050 aluminum alloys [21,22]. This indicates that the fatigue crack propagation in the 20% pre-stretched alloy plates deflected considerably over a wide range of angles between the adjacent grains and within each grain.

## 4. Discussion

The results in Figure 3, Figure 4 and Figure 5 show that the pre-tensile deformation did not have a significant influence on the shape, size, quantity, and distribution of the second-phase particles in the annealed 7005 aluminum plate, whereas the long axis of the thin strip grains was observed to be slightly elongated and the grain thickness was slightly reduced. The pre-stretching-induced plastic deformation had no obvious effect on the morphology, quantity, and distribution of the large excess α-Al(FeMn)Si particles (indicated by an arrow in Figure 3a), the sub-micron-sized dispersoids, and the MgZn_2_ precipitates (Figure 3b), because of the soft matrix of the annealed 7005 aluminum plate. The deformation resistance of the annealed alloy plate was remarkably low, and the yield strength of the alloy plate was only 123 MPa. The grain thickness slightly decreased and the grain length increased after a pre-stretching of <3% when the pre-tensile loading direction was consistent with the direction of the thin strip grain length. The yield strength of the alloy plate significantly increased from 183 to 269 MPa as the pre-tensile deformation increased from 3% to 20% because of the strain-hardening effect. The strengthened matrix impeded the modification of the grain shape, which ensured that the grain morphology and grain size remained unchanged with increasing pre-tensile deformation.

The increase in pre-tensile deformation results in dislocation multiplication, an increase in the number of dislocations, and the offsetting of unlike dislocations and dislocation annihilation that occurs when the dislocation slides out of the crystal surface. The primary reason why the strength and microhardness of the alloy plate improve with the increase of tensile deformation at room temperature is the increase of dislocation density, which is the essence of the strain-hardening mechanism. The variation of mechanical properties also shows that dislocation density does not increase linearly with the increase of strain in alloy plates, and the increase of dislocation density is accompanied by the change of dislocation distribution in the alloy matrix. Based on the variations in the microhardness, strength, and elongation of the investigated alloy plate with increasing pre-tensile deformation, the yield strength and tensile strength of the alloy plate were found to increase from 123 MPa and 210 MPa to 218 MPa and 230 MPa, respectively, whereas the elongation decreased from 29% to 20% at a pre-tensile deformation of <5%. The dislocation multiplication rate was ascertained to be considerably higher than the dislocation extinction or dislocation annihilation, which increased the number of dislocations in the alloy matrix. Moreover, the alloy plate exhibited a significant strain-hardening effect because of the pre-tensile deformation of <5%. The dislocation multiplication rate decreased and was considerably higher than the dislocation extinction or dislocation annihilation, which increased the number of dislocations in the alloy matrix. Moreover, the alloy plate exhibited a significant strain-hardening effect because the pre-tensile deformation was <5%. At pre-tensile deformations of over 5%, the dislocation multiplication rate gradually decreased and the dislocation extinction or dislocation annihilation rate increased, which led to an increased dislocation density in the alloy matrix. Moreover, the increase in the strength of the alloy plate was impeded. The yield strength and tensile strength of the pre-stretched alloy plates reached maximum values of 269 MPa and 271 MPa, respectively, at the pre-tensile deformation of 20%. However the corresponding elongation decreased to 6.8%.

The fatigue life and fatigue performance under certain fatigue loading conditions can be determined by the density and distribution of dislocations in the aluminum matrix of the various pre-stretched 7005 aluminum plate specimens. Adequate plasticity of a metal is believed to block fatigue crack propagation and improve its LCF life, whereas high strength can inhibit crack nucleation and improve its HCF life [23]. The annealed 7005 aluminum plate was found to have a soft and ductile matrix with a microhardness and elongation of approximately 61 HV and ~29%, respectively. However, the second-phase particles in the alloy matrix were brittle. Significant differences in hardness, strength, ductility, and toughness were observed between the α-Al matrix and second-phase particles, which caused uneven loading and inhomogeneous deformation in the alloy plate. These results are not favorable for the fatigue performance of the alloy plate. Numerous dislocations were found in the 3% pre-stretched alloy matrix at room temperature, and the microhardness of the aluminum alloy increased to 62 HV owing to the effect of dislocation strengthening. Moreover, the yield strength and tensile strength of the alloy plate increased to 183 MPa and 221 MPa, respectively, which were 60 MPa and 10 MPa greater than those of the annealed aluminum alloy plate. The dislocation-strengthened matrix narrowed the differences between the α-Al matrix and second-phase particles and reduced the degree of mismatch between the α-Al matrix and second-phase particles, improving the integral-compatible deformation capability of the 7005 aluminum plate. Fatigue crack initiation was induced by PSBs on the surface of the 3%-pre-stretched alloy plate, not by the second-phase particles (Figure 9 and Figure 10). Additionally, the surface of fatigue crack propagation was rough with large fluctuations, and a fatigue striation space of ~0.98 μm was observed (Figure 11a). The fatigue cracks were deflected several times and at large angles during crack propagation, which weakened the stress concentration at the main fatigue crack front. However, the wide fatigue striation space indicates that the fatigue crack propagation resistance in the alloy matrix was relatively small. Because of the dislocation strengthening of the α-Al matrix, the fatigue life of the 3% pre-stretched alloy plate was prolonged to 2.00 × 10^5^ cycles, which was 16% greater than that of undeformed annealed alloy plate (1.86 × 10^5^ cycles).

The dislocation density in the alloy matrix further increased as the pre-tensile deformation increased to 5%, and the alloy plate matrix was additionally strengthened owing to the effect of dislocation strengthening. The microhardness of the aluminum matrix increased to 61.4 HV, and the yield strength and tensile strength increased to 218 MPa and 230 MPa, respectively. The differences in microhardness and strength between the α-Al matrix and second-phase particles were further diminished. Moreover, the degree of mismatch between the α-Al matrix and second-phase particles was further reduced, and the integral-compatible deformation capability of the 7005 aluminum plate was further improved [24,25,26,27]. This increased the fatigue crack initiation resistance and facilitated an increase in the fatigue life of the alloy plate. However, the dislocation density and distribution in the adjacent grains were considerably different because of the asynchrony of dislocation sliding in the grains with various orientations of the α-Al matrix at the early stage of tensile deformation (Figure 5b and Figure 6a). Uneven deformation or the various dislocation densities in the grains slightly weakened the integral-compatible deformation capability of the alloy plates; moreover, the fatigue cracks were readily initiated at the grain boundaries on the surface of the alloy plate (Figure 9b), which reduced the fatigue crack initiation resistance and shortened the fatigue life of the alloy plates; this is associated with the fatigue crack initiation stage. In addition, the fatigue crack propagation resistance of the α-Al matrix was continually increased owing to the increased number of strengthened dislocations in the α-Al matrix; moreover, the surface of the fatigue crack propagation became rougher with larger fluctuations than that of the 3% pre-stretched alloy plate, and the fatigue striation space decreased to 0.84 μm (Figure 11b). This signified the deflection of fatigue cracks at larger angles during crack propagation, which weakened the stress concentration at the main fatigue crack front. Therefore, the fatigue life of the alloy plates associated with the fatigue crack propagation stage was extended. Furthermore, the uneven deformation or the various dislocation densities of the grains resulted in a high-density entangled dislocation in each grain, which had a bridging effect on the fatigue crack propagation. Therefore, fatigue crack propagation was facilitated, the fatigue crack propagation resistance was reduced, and the fatigue life of the alloy plate corresponding to the fatigue crack propagation stage was shortened. Overall, the synthetic result on aluminum matrix strengthening and uneven deformation of the grains in the 5% pre-stretched alloy plate shortened the fatigue life of the alloy plate slightly. Moreover, the fatigue crack propagation resistance was increased by matrix strengthening, and the fatigue life of the alloy plate was prolonged slightly during the fatigue crack propagation stage. The synergy between these shortening and prolonging aspects played a key role in the fatigue life of the alloy plate, which was shortened slightly to 1.82 × 10^5^ cycles with an increase in the pre-tensile deformation of the alloy plate from 3% to 5%. In conclusion, the fatigue life of the pre-stretched aluminum alloy plate is mainly dependent on the three aspects discussed henceforth. First, the resistance to fatigue crack propagation was increased by the strengthened matrix owing to the increase in dislocation density, which enabled the improvement in fatigue performance and the extension in fatigue life of the alloy plates. Second, the dislocation-strengthened α-Al matrix generated by the increasing dislocation density assisted in closing the gap or decreasing the degree of mismatch between the α-Al matrix and second-phase particles, which could improve the integral-compatible deformation capability of the 7005 aluminum plate and elevate the fatigue crack initiation resistance. Moreover, this had a positive effect on the improvement in fatigue performance and the prolongation of the fatigue life of the alloy plate. Additionally, heterogeneous dislocation distribution in the aluminum alloy matrix due to uneven deformation weakened the integral-compatible deformation capability of the aluminum plate and decreased the resistance to fatigue crack initiation. Moreover, the high-density entangled dislocation in the grains had a bridging effect on the fatigue crack propagation, which facilitated fatigue crack propagation or decreased the fatigue crack propagation resistance of the alloy plate. Consequently, this hindered the improvement in fatigue performance and the prolongation of the fatigue life of the alloy plate.

Upon increasing the pre-tensile deformation of the alloy plate to 8%, the dislocation density in the matrix further increased, and the plastic deformation spread throughout the alloy matrix. The dislocation densities in the various grain orientations of the alloy plate were almost identical, and a homogeneous dislocation distribution in the grains was observed (Figure 5c and Figure 6b). The microhardness of the aluminum matrix increased by 3.9 HV, and the yield strength and tensile strength of the aluminum alloy plate increased to 226 MPa and 235 MPa, respectively. This narrowed the gap and decreased the degree of mismatch between the α-Al matrix and second-phase particles. More importantly, the dislocation distribution in the various grains was approximately similar, and the dislocation distribution within each grain was homogeneous, which significantly enhanced the fatigue crack initiation resistance of the alloy plate. Therefore, the fatigue life of the alloy plate corresponding to the fatigue crack initiation stage was significantly prolonged. Fatigue cracks were primarily initiated at the PSBs on the alloy plate surfaces and extended radially from their surfaces into their interior (Figure 9c). Furthermore, the resistance to fatigue crack propagation was increased by the additionally strengthened matrix owing to the increase in dislocation density of the alloy plate; moreover, the fatigue striations in fatigue crack propagation were clearly refined, and the fatigue striation space was reduced to 0.78 μm (Figure 11c). However, the surface of the fatigue crack propagation became flatter without obvious fluctuations compared to that of the 5% pre-stretched alloy plate (Figure 11c), because the dislocation density and dislocation distribution in the various oriented grains in the alloy plate were almost similar. This signifies the deflection of the fatigue cracks at smaller angles. However, it occurred several times during crack propagation, which weakened the stress concentration at the main fatigue crack front. In conclusion, for the 8% pre-stretched aluminum alloy plate, the homogenous enhancement in the alloy matrix due to the high density and homogenous distribution of dislocations in the various oriented grains of the alloy plate not only increased its fatigue crack initiation resistance but also its fatigue crack propagation resistance. Therefore, the fatigue life of the alloy plate at both the fatigue crack initiation and propagation stages was prolonged to 5.21 × 10^5^ cycles.

Therefore, the fatigue life of the alloy plates associated with the fatigue crack propagation stage was extended. The surface of the fatigue crack growth zone on the fatigue fracture surface was relatively flat without obvious fluctuations, because the number and distribution of dislocations in the grains with different orientations were nearly identical, indicating the small degree of deflection of the fatigue crack growth in the 8% pre-stretched alloy plate.

The microhardness of the aluminum matrix increased to 70.1 HV and 73.9 HV when the pre-tensile deformation increased to 12% and 16%, respectively (Figure 7a); this indicates that the dislocation density in the alloy plate matrix continued to increase. Moreover, the yield strength of the alloy plate increased to 246 MPa and 261 MPa, respectively, and the elongation of the alloy plate decreased to 16.8% and 10.1%, respectively. It is worth noting that the yield strength of the alloy plate was nearly identical to the tensile strength, and the ratio of yield strength to tensile strength of the 12% and 16% pre-stretched specimens was approximately 0.99. Dislocation cells and sub-grains were clearly visible in the 12% pre-stretched alloy plate grains owing to the rearrangement of the high-density dislocations in the grains (Figure 5e and Figure 6d), indicating the heterogeneous distribution of high-density dislocations in each grain. This heterogeneous distribution creates a stress concentration in the local region of the alloy plate, which provides favorable locations for fatigue crack initiation and reduces the fatigue crack initiation resistance of the alloy plate. Fatigue crack initiation was always found to occur in the PSBs on the alloy plate surfaces (Figure 9g). The heterogeneous distribution of the dislocations in each grain decreased the fatigue life corresponding to the fatigue crack initiation stage of the alloy plate. Upon increasing the pre-tensile deformation to 16%, the dislocation density in the alloy plate grains further increased, whereas the dislocation distribution in each grain and among the grains tended to be homogeneous (Figure 5f and Figure 6e). Fatigue cracks were mainly initiated at the PSBs on the surface of the alloy plate (Figure 9e). Therefore, the fatigue life corresponding to the fatigue crack initiation stage of the 16% pre-stretched alloy plate was slightly longer than that of the alloy plate pre-stretched by 12% owing to the more homogeneous dislocation distribution in each grain. Moreover, the fatigue fracture surface of the alloy plate was relatively smooth, and the tear edge fluctuation was small (Figure 11d,e), indicating that the main fatigue crack exhibited several small-angle deflections during crack propagation. The fatigue striation space further reduced to 0.64 μm and 0.52 μm in the 12% and 16% pre-stretched alloy plates, respectively. In addition, certain secondary cracks were observed in the propagation region of the fatigue fracture surface, which released the stress concentration at the main fatigue crack front and facilitated a reduction in the fatigue crack propagation rate [28]. This indicates that the fatigue crack propagation resistance of the dislocation-strengthened α-Al matrix was further increased, and the fatigue life of the alloy plate corresponding to the fatigue crack propagation stage was prolonged. In addition, the dislocation cells and sub-grains resulting from the heterogeneously distributed high-density dislocations within the grains had a bridging effect on the fatigue crack propagation, which improved the fatigue crack propagation rate, reduced the fatigue crack propagation resistance, and shortened the fatigue life of the alloy plate corresponding to the fatigue crack propagation stage.

Two factors were emphasized when conducting the comprehensive analysis of the impact of pre-stretching-induced dislocations on the fatigue performance of the alloy plate. First, the fatigue life of the alloy plate corresponding to the initiation and propagation of fatigue cracks was slightly shortened owing to the inhomogeneous distribution of dislocations in each grain of the aluminum matrix. Second, the dislocation-strengthened matrix due to the increase in dislocation density of the alloy plate reduced the degree of mismatch between the α-Al matrix and second-phase particles, improved the integral-compatible deformation capability, and increased the fatigue crack propagation resistance, which facilitated extension of the fatigue life of the 7005 aluminum plate. These two factors synergistically contributed to determining the fatigue performance and fatigue life of the alloy plate. The fatigue life of the 12% pre-stretched alloy plate (5.12 × 10^5^ cycles) was slightly lower than that of the 8% pre-stretched plate (5.21 × 10^5^ cycles). However, the fatigue life of the 16% pre-stretched alloy plate was extended to 5.44 × 10^5^ cycles, which was approximately 4.4% greater than that of the 8% pre-stretched alloy plate.

Upon increasing the pre-tensile deformation to 20%, the increase in microhardness of the alloy plate was only 0.2 HV, and the yield strength and tensile strength of the alloy plate reached their maximum values of 269 MPa and 271 MPa, respectively. This indicates that the dislocation density in the alloy plate was almost saturated, and the dislocations that migrated from the crystal surface or the dislocation offset inside the matrix were in dynamic equilibrium with the dislocation multiplication. The dislocation density and dislocation distribution in the various grains and within each grain were nearly identical (Figure 5g and Figure 6f). The optimal dislocation-strengthened matrix minimized the differences and the degree of mismatch between the α-Al matrix and second-phase particles, which significantly improved the integral-compatible deformation capability of the 7005 aluminum plate. The fatigue crack initiation resistance of the alloy plate was dramatically improved, and the fatigue life of the alloy plate corresponding to the fatigue crack initiation stage was significantly extended. The characteristic of the fatigue cracks that were initiated at the PSBs of the alloy plate surface and radially propagated from a crack source deep in its interior was more evident (Figure 9f). Moreover, the resistance of the α-Al matrix to the fatigue crack propagation significantly increased owing to the most significant dislocation strengthening effect among the specimens, and the fatigue striation space was reduced to 0.4 μm. The surface morphology featuring the fatigue crack propagation indicated that this characteristic in the 20% pre-stretched alloy plate was deflected by several times and at large angles. In conclusion, the initiation and propagation resistances to fatigue cracks of the alloy plate were dramatically improved owing to the highest density and the most homogeneous distribution of dislocations in the α-Al matrix among the specimens, which considerably improved the integral-compatible deformation capability of the 7005 aluminum plate. Consequently, the fatigue lives corresponding to the initiation and propagation stages of fatigue cracks in the 20% pre-stretched alloy plate were considerably extended. Furthermore, a fatigue life over 1.06 × 10^6^ cycles for the alloy plate was obtained, which was 5.7 times and 5.3 times greater than those of the undeformed and 3% pre-stretched alloy plates, respectively.

Previous research has suggested that the fatigue life corresponding to crack initiation accounts for approximately 90% of the entire fatigue life of smooth metallic specimens, with the leftover part involving the fatigue life related to crack propagation [29]. Therefore, the contribution of pre-tensile deformation to the fatigue life of the annealed 7005 aluminum alloy plate can be semi-quantitatively calculated as follows:

(1) $$\lambda =0.9\lambda_{i(90\%)}+0.1\lambda_{p(10\%)}$$

where *λ* is the impact factor of the pre-tensile deformation on the fatigue life of the pre-stretched alloy plate, and *λ_i_* and *λ_p_* are the components of the impact factor corresponding to the initiation and propagation resistances to fatigue cracks of the pre-stretched alloy plates, respectively. *λ_i_* and *λ_p_* can be calculated based on the dislocation density and dislocation distribution effects. Notations of “+” and “−” were used to represent positive and negative impacts on the fatigue crack initiation resistance or fatigue crack propagation resistance of the alloy plate; a greater number of “+” or “−” indicates greater contributions. The calculated impact factors of the pre-tensile deformations on the fatigue life of the annealed 7005 aluminum alloy plate according to Equation (1) are shown in Table 1.

## 5. Conclusions

(1) With increasing pre-tensile deformation, small changes are observed on the secondary particles while the shape, size, quantity, and distribution of the second-phase particles showed no obvious changes, whereas the thickness of the thin strip grain slightly decreased when the pre-tensile deformation of the annealed 7005 aluminum alloy plate was increased to 20%.
(2) The mechanical properties increased with the increase of pre-tensile deformation, and the yield strength achieved 271 MPa with 20% pre-tensile deformation compared with 123 MPa in the undeformed annealed condition
(3) Generally, the fatigue life and performance were improved with the increasing of pre-tensile deformation and the optimal fatigue life of ~1.11 × 106 cycles was obtained in alloy with 20% pre-tensile deformation, which is 5.7 times that of undeformed alloy.
(4) The fatigue performance was strongly related to the dislocation density and its distribution between/within grains. Although the dislocation density gradually increased with increasing pre-tensile deformation, the increased heterogenous distribution between/within grains observed from 3% to 5% and from 8% to 16% resulted in two plateaux for fatigue life. The dislocation density reached a maximum with homogeneous distribution in the various grains and within each grain at a pre-tensile deformation of 20%, leading to the best fatigue performance.

## Figures and Tables

**Table 1:** Calculated impact factors of pre-tensile deformations on fatigue life of annealed 7005 aluminum alloy plate according to Equation (1).

Impact Factor Calculations Pre-Tensile Deformation	Effect of Dislocation Density		Effect of Dislocation Distribution		*λ*
	*λ_i_*	*λ_p_*	*λ_i_*	*λ_p_*	
3%	+++	+			2.8
5%	++++	++	−	−	2.8
8%	+++++	++	+	+	5.7
12%	+++++++	++++	−	−−	5.6
16%	++++++++	+++++	−−		5.9
20%	++++ ++++	+++++	+	++	8.8

## Data Availability

The data presented in this study are available on request from the corresponding author.
